# Dietary apple pectic oligosaccharide improves gut barrier function of rotavirus-challenged weaned pigs by increasing antioxidant capacity of enterocytes

**DOI:** 10.18632/oncotarget.21367

**Published:** 2017-09-28

**Authors:** Xiangbing Mao, Xiangjun Xiao, Daiwen Chen, Bing Yu, Jun He, Hao Chen, Xuechun Xiao, Junqiu Luo, Yuheng Luo, Gang Tian, Jianping Wang

**Affiliations:** ^1^ Animal Nutrition Institute, Sichuan Agricultural University, Wenjiang District, Chengdu, 611130, People's Republic of China; ^2^ Key Laboratory of Animal Disease-Resistance Nutrition, Chinese Ministry of Education, Chengdu, 611130, People's Republic of China

**Keywords:** apple pectic oligosaccharide, gut barrier function, rotavirus, weaned piglets, antioxidant capacity

## Abstract

Rotavirus can lead to decreasing gut barrier function and diarrhea of children and young animals. Apple pectic oligosaccharide treatment reduced diarrhea in rotavirus-infected piglets. This study was conducted to explore whether apple pectic oligosaccharide administration could protect gut barrier function of piglets against rotavirus infection. A total of 28 crossbred weaned barrows were allotted into 2 treatments fed the diets supplementing 0 and 200 mg/kg apple pectic oligosaccharide. Half of pigs in each diet treatment were challenged by rotavirus on d 15. The whole duration of this experiment is 18 days. Rotavirus challenge increased average diarrhea index, and impaired microbiota in cecal digesta, and histology, expressions of tight-junction proteins, mucins and glucagon like peptide-2 concentrations, antioxidant capacity, endoplasmic reticulum stress, autophagy and apoptosis in jejunal mucosa of piglets. However, dietary apple pectic oligosaccharide supplementation relieved effects of rotavirus challenge on diarrhea, gut health, and antioxidant capacity, endoplasmic reticulum stress, autophagy and apoptosis of jejunal mucosa in piglets. These results suggest that apple pectic oligosaccharide administration can prevent diarrhea and damage of gut barrier function via improving antioxidant capacity that might reduce endoplasmic reticulum stress, autophagy and apoptosis of intestinal epithelial cells in rotavirus-infected piglets.

## INTRODUCTION

Pectic oligosaccharide (POS) is one of functional oligosaccharides, which mainly consists of pectic disaccharide and trisaccharide that contain galacturonic acid. Some studies have shown that dietary hawthorn POS supplementation can regulate lipid metabolism and antioxidant capacity in mice [1, 2], and sugar beet POS can improve intestinal microflora of human and pigs in the *in vitro* experiments [3]. Our previous studies also indicated that dietary apple pectic oligosaccharide (APOS) supplementation could improve growth performance, antioxidant capacity, intestinal flora structure and jejunal mucosal morphology in rats [4], and might improve the immunity in piglets [5].

As the first barrier of body, gut play a critical important role for health and growth of human and animals [6]. However, as known to all, intestine development in children and young animals is incomplete, so its functions and structures are easily impaired by all kinds of pathogens, such as rotavirus (RV). RV infection induces diarrhea via damaging the intestinal health in children and young animals [7, 8]. And our previous studies also reported that RV challenge could impair growth and health of piglets partially through affecting gut barrier function [9–12]. It is extensively focused on how to prevent and cure the impairment of RV infection.

RV can induce the oxidative stress of piglets (especially small intestine), which promotes the endoplasmic reticulum stress and autophagy in gut epithelial cells [11–13]. This could be one of the important reasons that RV results in gut dysfunction and diarrhea of humans and animals. On basis of POS physiological function that may improve antioxidant capacity and gut mucosal structure, it is possible that POS pre-administration alleviated the effect of RV on intestinal health.

Therefore, the aim of this study was to test the hypothesis that dietary APOS supplementation could protect gut mucosal barrier function against RV infection, which would effectively attenuate diarrhea. The possible mechanism was also studied.

## RESULTS

### Diarrhea and non-structural protein 4 (NSP4) concentration of jejunal mucosa

Following RV infection, average diarrhea index and NSP4 concentration of jejunal mucosa was increased in weaned pigs (*P* < 0.05; Table 1). However, dietary APOS supplementation could significantly attenuate diarrhea, and decrease NSP4 concentration of jejunal mucosa in weaned pigs challenged by RV (*P*< 0.05; Table 1).

**Table 1:** The effect of dietary APOS supplementation and/or RV challenge on the diarrhea and the NSP4 concentration of jejunal mucosa in weaned pigs (n=7)

	RV -		RV +		*P*-value		
	CON	POS	CON	POS	POS	RV	POS×RV
Average diarrhea index	0.11±0.07^C^	0.00±0.00^C^	1.51±0.26^A^	0.74±0.24^B^	<0.05	<0.05	0.09
NSP4 (pg/mg protein)	48.91±3.76^C^	46.81±3.40^C^	170.13±13.78^A^	119.62±14.23^B^	<0.05	<0.05	<0.05

^#^ RV -, infusing the essential medium; RV +, infusing the porcine rotavirus; CON, basal diet; POS, POS-supplemented diet.

^A, B, C^ In the same row, values with different letter superscripts mean significant difference (*P*<0.05).

### Histology of jejunal mucosa

**Figure 1:**
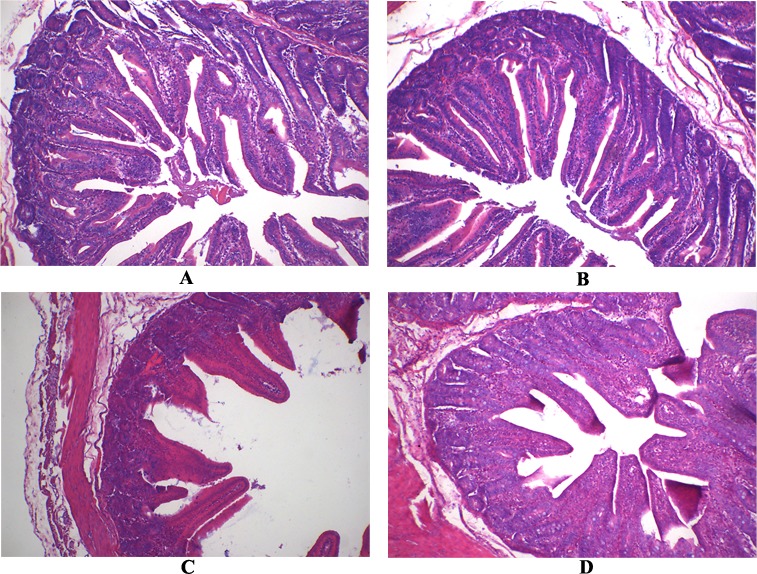
The jejunal mucosal morphology of the weaned pigs **(A)** The pig fed the basal diet and orally infused with the sterile essential medium; **(B)** the pig fed the APOS supplementing diet and orally infused with the sterile essential medium; **(C)** the pig fed the basal diet and orally infused with rotavirus; **(D)** the pig fed the APOS supplementing diet and orally infused with rotavirus. (Original magnification, 100 ×).

Following RV challenge, villus height and villus height:crypt depth were decreased in jejunal mucosa of weaned pigs (*P* < 0.05; Table 2 and Figure 1). However, supplementing APOS in diets increased villus height and villus height:crypt depth of jejunal mucosa in weaned pigs (*P* < 0.05; Table 2 and Figure 1). Moreover, in weaned pigs infected by RV, dietary APOS supplementation could alleviate the effect of RV challenge on villus height and villus height:crypt depth of jejunal mucosa (*P* < 0.05; Table 2 and Figure 1).

**Table 2:** The effect of dietary APOS supplementation and/or RV challenge on the intestinal morphology in the jejunum of weaned pigs (n=7)

	RV -		RV +		*P*-value		
	CON	POS	CON	POS	POS	RV	POS×RV
Villus height (μm)	179.22±2.45^A^	196.55±5.23^A^	156.73±7.11^B^	177.80±9.77^A^	<0.05	<0.05	0.78
Crypt depth (μm)	94.31±4.37	95.71±4.46	105.26±3.26	91.84±4.58	0.17	0.42	0.09
Villus height:crypt depth	1.92±0.08^A^	2.08±0.12^A^	1.49±0.07^B^	1.95±0.08^A^	<0.05	<0.05	0.13

^#^ RV -, infusing the essential medium; RV +, infusing the porcine rotavirus; CON, basal diet; POS, POS-supplemented diet.

^A, B^ In the same row, values with different letter superscripts mean significant difference (*P*<0.05).

### Expressions of tight junction proteins and concentrations of mucins in jejunal mucosa

**Figure 2:**
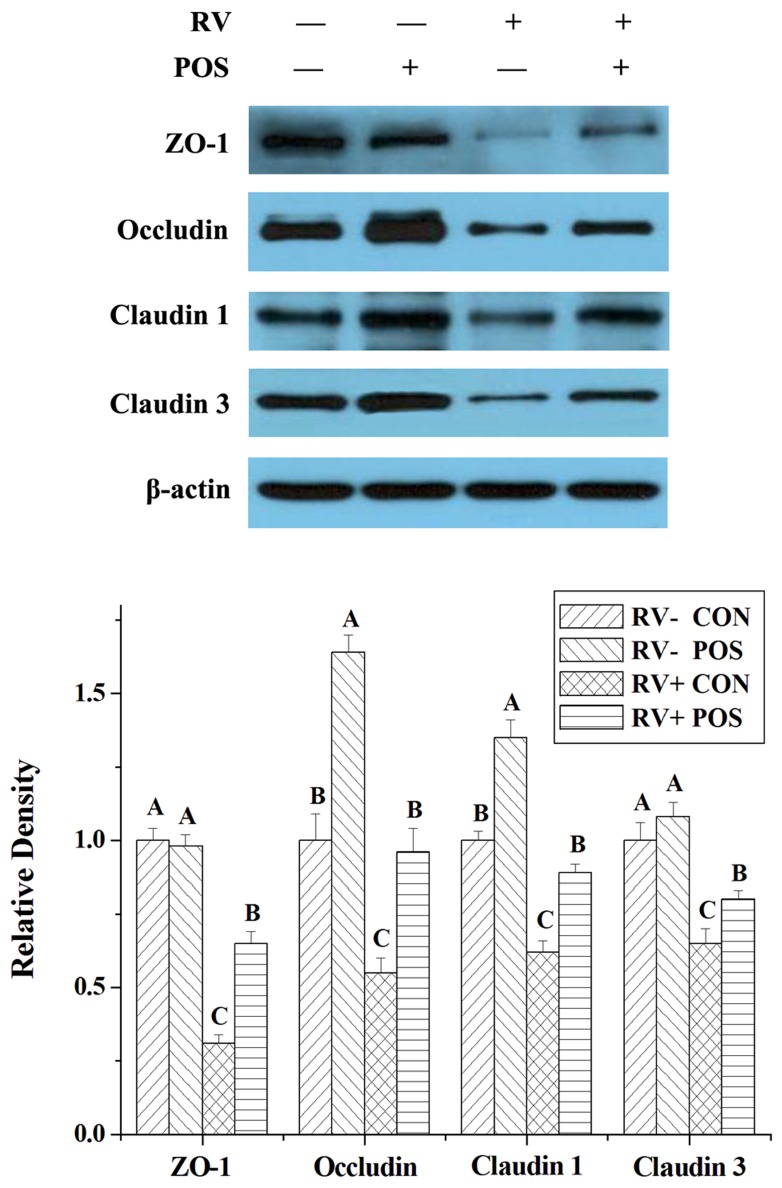
The effect of dietary APOS supplementation and/or RV challenge on the levels of ZO-1, occludin, claudin 1 and claudin 3 in the jejunal mucosa of weaned pigs Representative Western blots for ZO-1, occludin, claudin 1, claudin 3 and β-actin in the jejunal mucosa of weaned pigs were shown. Results were expressed as the amount of ZO-1, occludin, claudin 1 and claudin 3 to β-actin in each treatment as a ratio of the other pigs to the unchallenging pigs fed basal diet. Values are means ± SE; n = 7. Values with different letters are significantly different (*P* < 0.05).

RV infection decreased expressions of zonula occludens 1 (ZO-1), occludin, claudin 1 and claudin 3, and concentrations of mucin 1 and mucin 2 in jejunal mucosa of weaned pigs (*P* < 0.05; Table 3 and Figure 2). However, supplementing APOS in diets stimulated expressions of ZO-1, occludin, claudin 1 and claudin 3, and enhanced concentrations of mucin 1 and mucin 2 in jejunal mucosa of weaned pigs (*P* < 0.05; Table 3 and Figure 2). Furthermore, in the piglets challenged by RV, the effect of RV infusion on ZO-1, occludin, claudin 1, claudin 3, mucin 1 and mucin 2 of jejunal mucosa could be relieved by dietary APOS supplementation (*P* < 0.05; Table 3 and Figure 2).

**Table 3:** The effect of dietary APOS supplementation and/or RV challenge on concentrations of mucins in the jejunal mucosa of weaned pigs (n=7)

	RV -		RV +		*P*-value		
	CON	POS	CON	POS	POS	RV	POS×RV
Mucin 1 (U/mg protein)	175.15±9.03^B^	207.48±10.22^A^	116.25±7.93^C^	161.06±9.81^B^	<0.05	<0.05	0.51
Mucin 2 (ng/mg protein)	40.53±1.43^B^	48.11±1.78^A^	33.52±1.16^C^	39.73±1.86^B^	<0.05	<0.05	0.67

^#^ RV -, infusing the essential medium; RV +, infusing the porcine rotavirus; CON, basal diet; POS, POS-supplemented diet.

^A, B, C^ In the same row, values with different letter superscripts mean significant difference (*P*<0.05).

### Bacteria, pH value and volatile fatty acid (VFA) concentrations in cecal digesta

After RV infusion, *Lactobacillus* and *Bifidoba-cterium* populations were decreased (*P* < 0.05), *E. coli* and total bacteria populations were increased (*P* < 0.05), the pH value were enhanced (*P* < 0.05), and acetate and total VFA concentrations were reduced (*P* < 0.05) in cecal digesta of weaned pigs (Table 4). Dietary APOS supplementation could increase populations of *Lactobacillus*, *Bifidobacterium* and total bacteria (*P* < 0.05), decrease *E. coli* population (*P* < 0.05), reduce pH value (*P* < 0.05), and enhance concentrations of acetate and total VFA (*P* < 0.05) in cecal digesta of weaned pigs (Table 4). Additionally, in the piglets challenged by RV, supplementing APOS in diets could alleviate the effect of RV infusion on populations of *Lactobacillus*, *Bifidobacterium* and *E. coli*, pH value, and concentrations of acetate and total VFA in cecal digesta (*P* < 0.05; Table 4).

**Table 4:** The effect of dietary APOS supplementation and/or RV challenge on bacteria, pH value and concentrations of volatile fatty acids (VFA) in the cecal digesta of weaned pigs (n=7)

	RV -		RV +		*P*-value		
	CON	POS	CON	POS	POS	RV	POS×RV
Bacterium populations (log_10_(copies/g))							
*Lactobacillus*	8.16±0.02^C^	8.48±0.01^A^	8.02±0.02^D^	8.30±0.02^B^	<0.05	<0.05	0.19
*Bifidobacterium*	3.14±0.03^C^	4.10±0.04^A^	2.38±0.03^D^	3.50±0.04^B^	<0.05	<0.05	<0.05
*E. coli*	7.91±0.04^C^	7.73±0.03^D^	8.35±0.02^A^	8.18±0.03^B^	<0.05	<0.05	0.99
Total bacteria	11.81±0.06^C^	11.96±0.03^B^	11.87±0.02^BC^	12.08±0.02^A^	<0.05	<0.05	0.45
pH value	5.61±0.05^B^	5.49±0.02^B^	6.06±0.06^A^	5.62±0.02^B^	<0.05	<0.05	<0.05
VFA concentrations (mg/g)							
Acetate	3.98±0.29^B^	5.19±0.29^A^	3.68±0.26^C^	3.83±0.24^B^	<0.05	<0.05	0.43
Propionate	2.08±0.19	2.59±0.18	2.00±0.16	2.16±0.15	0.14	0.06	0.31
Butyrate	0.72±0.09	0.83±0.09	0.60±0.08	0.76±0.07	0.13	0.31	0.78
Total VFA	6.79±0.49^B^	8.62±0.49^A^	6.29±0.45^C^	6.62±0.41^B^	<0.05	<0.05	0.12

^#^ RV -, infusing the essential medium; RV +, infusing the porcine rotavirus; CON, basal diet; POS, POS-supplemented diet.

^A, B, C, D^ In the same row, values with different letter superscripts mean significant difference (*P*<0.05).

### Glucagon like peptide-2 (GLP-2) level and antioxidant capacity in jejunal mucosa

Via RV challenge, GLP-2 concentration of jejunal mucosa was reduced (*P* < 0.05), and total antioxidant capacity (T-AOC) of jejunal mucosa was inhibited (*P* < 0.05), but malondialdehyde (MDA) concentration of jejunal mucosa was increased (*P* < 0.05) in weaned pigs (Table 5). However, dietary APOS supplementation could enhanced T-AOC (*P* < 0.05), and decreased MDA levels (*P* < 0.05) in jejunal mucosa of weaned pigs (Table 5). The results also showed that, in the pigs challenged by RV, dietary APOS supplementation could improve the effect of RV infection on T-AOC and MDA levels in jejunal mucosa (*P* < 0.05; Table 5).

**Table 5:** The effect of dietary APOS supplementation and/or RV challenge on GLP-2 levels and antioxidant capacity in the jejunal mucosa of weaned pigs (n=7)

	RV -		RV +		*P*-value		
	CON	POS	CON	POS	POS	RV	POS×RV
GLP-2 (pg/mg protein)	202.03±15.04^A^	194.16±21.47^A^	151.88±3.90^B^	122.72±16.16^B^	0.24	<0.05	0.50
MDA (nmol/mg protein)	0.49±0.01^C^	0.46±0.01^C^	0.73±0.01^A^	0.55±0.01^B^	<0.05	<0.05	<0.05
T-AOC (U/mg protein)	0.42±0.01^A^	0.45±0.02^A^	0.35±0.02^B^	0.42±0.01^A^	<0.05	<0.05	0.17

^#^ RV -, infusing the essential medium; RV +, infusing the procine rotavirus; CON, basal diet; POS, POS-supplemented diet.

^A, B^ In the same row, values with different letter superscripts mean significant difference (*P*<0.05).

### Levels of endoplasmic reticulum stress (ERS)-, autophagy- and apoptosis-relative proteins in jejunal mucosa

**Figure 3:**
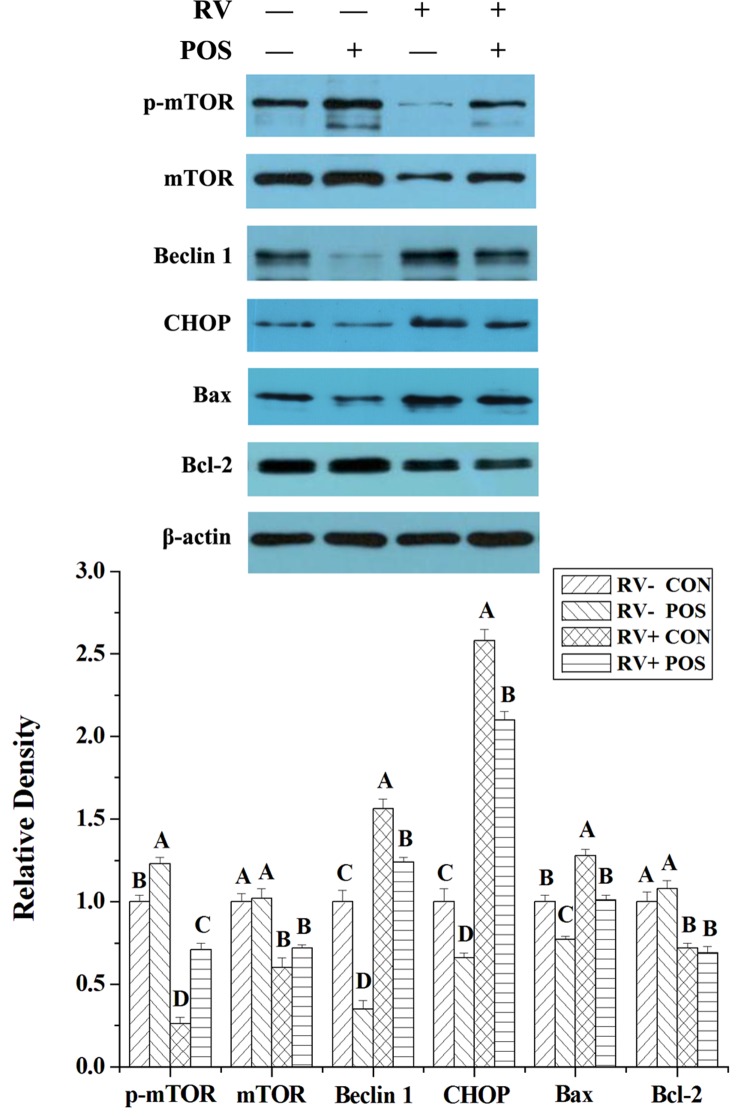
The effect of dietary APOS supplementation and/or RV challenge on the levels of p-mTOR, mTOR, Beclin 1, CHOP, Bax and Bcl-2 in the jejunal mucosa of weaned pigs Representative Western blots for p-mTOR, mTOR, Beclin 1, CHOP, Bax, Bcl-2 and β-actin in the jejunal mucosa of weaned pigs were shown. Results were expressed as the amount of p-mTOR, mTOR, Beclin 1, CHOP, Bax and Bcl-2 to β-actin in each treatment as a ratio of the other pigs to the unchallenging pigs fed basal diet. Values are means ± SE; n = 7. Values with different letters are significantly different (*P* < 0.05).

For determining the possible effect of ERS, autophagy and apoptosis, the levels of some proteins in these pathways, such as phosphorylated mammalian target of rapamycin (p-mTOR; Ser^2448^), mTOR, Beclin 1, CCAAT/enhancer-binding protein homologous protein (CHOP), B-cell lymphoma/leukaemia-2- associated X protein (Bax) and B-cell lymphoma/leukaemia-2 (Bcl-2), were analyzed by Western Blot. Following RV infection, levels of p-mTOR, mTOR and Bcl-2 were reduced (*P* < 0.05), and levels of Beclin 1, CHOP and Bax were increased (*P* < 0.05) in jejunal mucosa of weaned pigs (Figure 3). Dietary APOS supplementation could stimulate p-mTOR (*P* < 0.05), and inhibit levels of Beclin 1, CHOP and Bax (*P* < 0.05) in jejunal mucosa of weaned pigs (Figure 3). Furthermore, in the piglets challenged by RV, dietary APOS supplementation could improve the effect of RV infection on levels of p-mTOR, Beclin 1, CHOP and Bax in jejunal mucosa (*P* < 0.05; Figure 3).

## DISCUSSION

RV is well known as the pathogen that impairs the health mainly via inducing the serious diarrhea in young animals and children [7–8]. In this study, RV challenge led to the diarrhea, and destroyed gut barrier function in weaned pigs (Tables 1-4 and Figure 1-2), which was consistent with the previous studies [9–12]. Furthermore, the present study also showed that the NSP4 level was enhanced by RV infusion in jejunal mucosa of weaned pigs (Table 1). Thus, the model of RV infection was successful in this work. The important finding of the current study is that supplementing APOS in diets could relieve the diarrhea induced by RV challenge in weaned pigs (Table 1).

Gut barrier function plays a critical role for maintaining the intestinal health. Our previous study have shown that dietary POS supplementation could improve the immunological barrier of jejunal mucosa via regulating the levels of RV antibody, sIgA and some cytokines in the weaned pigs infected by RV [5]. This study reported that APOS administration could reduce the NSP4 level, related to the RV replication and pathogenicity [14], in jejunal mucosa of weaned pigs challenged by RV (Table 1). These could demonstrated that dietary APOS supplementation improved the immunological function of gut mucosa, and then inhibited the RV replication in jejunum of weaned pigs.

Our current studies showed that, under normal or RV-challenge conditions, APOS administration increased population of effective microorganisms, decreased population of harmful microorganisms, and enhanced VFA concentrations in cecal digesta of weaned pigs (Table 4). As description of many previous studies, intestinal microbiota and their relative metabolites (including VFA) play an important role for human and animal health as an indispensable part of gut barrier function [15, 16]. It is possible that supplementing APOS in diets alleviating the diarrhea induced by RV challenge should be relative with improving gut microbiota in piglets.

Besides immunological barrier and microbial, mucosal-epithelial integrity, intercellular junctions between the epithelial cells, and mucus gel layer are also important to protect gut against the pathogens [6, 17]. Intestinal mucosal integrity is evaluated through analyzing mucosal surface area with morphology [18]. Mucins, produced by goblet cells in intestinal epithelial, are the main components of mucus gel layer [19]. Additionally, some transmembrane and nonmembrane proteins, such as ZO-1, occludin and claudins, mainly form the intercellular junctions between the epithelial cells [20]. The results of our study showed that APOS administration improved jejunal mucosal morphology, and enhanced expressions of ZO-1, occludin, claudin 1 and claudin 3, and concentrations of mucin 1 and mucin 2 of jejunal mucosa in weaned pigs infected by RV (Tables 2-3 and Figure 1-2), which illustrated that dietary APOS supplementation alleviating the RV infection could also be relative with the improvement of mucosal-epithelial integrity, intercellular junctions between the epithelial cells, and mucus gel layer.

The previous study in our lab have shown that RV infection inducing the damage of intestinal epithelia and IPEC-J2 cells could mainly be relative with cell apoptosis [12]. And RV inhibiting mucin production is derived from decreasing the number of intestinal epithelia and goblet cells [21]. GLP-2, a 33-amino acid proglucagon-derived peptide, is produced and secreted by gut enteroendocrine cells, which can promote intestinal growth and mucosal repair, increase epithelial cell proliferation, and inhibit enterocyte apoptosis [22, 23]. In the current study, RV infection significantly reduced GLP-2 concentration in jejunal mucosa of piglets, but APOS administration did not attenuate the effect of RV challenge on GLP-2 level (Table 5). Therefore, it is possible that RV infection damaging gut barrier function was, at least partially, due to the reducing GLP-2 production, and APOS supplementation in diets improving intestinal barrier function affected by RV challenge (including mucosal-epithelial integrity, intercellular junctions between the epithelial cells, and the mucus gel layer) was unrelated to GLP-2 production in piglets.

The redox status is vital for cellular survival and growth. The oxidative stress in cells will induce ERS and autophagy, which can lead to cell apoptosis [24, 25]. Autophagy is mediated by a complex molecular machinery, in which mTOR phosphorylation may inhibit the initiation step of macroautophagy, and Beclin 1 plays a critical role for autophagosomal formation and expansion [26, 27]. CHOP is one of specific transcription factors in ERS, and its stimulation is a kind of mechanism that ERS induces cell apoptosis [24, 28]. Apoptosis initiated at the mitochondria is regulated by the Bcl-2 family of proteins, including anti-apoptotic proteins (such as Bcl-2) and pro-apoptotic proteins (such as Bax) [25].

In our previous study, RV infection may decrease antioxidant capacity, induce ERS, and increase autophagy in IPEC-J2 cells and small intestine of piglets [11–13]. The present study showed that RV infection reduced T-AOC, increased the lipid peroxidation product (MDA), and affected expressions of p-mTOR, mTOR, Beclin 1, CHOP, Bax and Bcl-2 in jejunal mucosa of piglets (Table 5 and Figure 3), which could demonstrate that, besides affecting the enteroendocrine, RV may increase cell apoptosis via impairing antioxidant capacity, ERS and autophagy. Furthermore, in this study, APOS administration significantly relieved the effect of RV challenge on antioxidant capacity and expressions of p-mTOR, Beclin 1, CHOP and Bax in jejunal mucosa of piglets (Table 5 and Figure 3). Thus, this suggested that increasing antioxidant capacity that inhibited cell apoptosis through reducing ERS and autophagy could be an important reason that dietary APOS supplementation improved gut barrier function.

In summary, RV infection inducing diarrhea of piglets was relative with the damage of intestinal barrier function via the increase of cell apoptosis that was due to the decreasing GLP-2 level and antioxidant capacity. However, dietary APOS supplementation could attenuate diarrhea of piglets via inhibiting the effect of RV challenge on gut barrier function. Furthermore, we also found that POS administration decreased cell apoptosis possibly via decreasing RV multiplication, enhancing antioxidant capacity, and reducing ERS and autophagy in piglets infected by RV. These could be the important reasons of APOS treatment reducing diarrhea of RV-infected piglets. In addition, as a result of the high resemblance between humans and pigs (especially gut development and function), pigs are often used as an experimental animal model for humans. Based on the tissue resemblance between pigs and humans, this study will provide a possibility for APOS preventing diarrhea induced by RV infection in children.

## MATERIALS AND METHODS

### Animals and diets

The experimental protocol was approved the Animal Care Advisory Committee of Sichuan Agricultural University. Twenty-eight crossbred (Duroc × Large White × Landrace) barrows weaned at 21 d of age were individually housed in the metabolic cage (1.5 m × 0.7 m × 1.0 m). For all pigs, the experimental diets were fed 4 times daily at 0800, 1200, 1600 and 2000, and water was able to be freely accessed.

The experimental diets were formulated to approximately meet National Research Council-recommended nutrient requirements (NRC, 2012) for pigs weighing 7-11 kg [29], which is shown in Supplementary Table 1. The APOS product was obtained from Hebei Kena Biological Technology Co. Ltd. (Hebei, China). The contents of APOS and corn starch are 30% and 70% in this product, respectively.

### Experimental design, porcine rotavirus preparation and sample collection

Following acclimatization (3 days), based on origin of litters and initial body weight, all piglets (approximately 7.54 kg) were allotted randomly to one of two treatments fed by diets with or without 200 mg/kg APOS product (n = 14) for 18 d. At d 15, all piglets were infused 5 mL of a 100 mmol/L sterile sodium bicarbonate solution. After 20 min, half of pigs on each treatment were orally infused with 4 mL (10^6^ Tissue culture infective dose 50 (TCID50)/mL) of porcine RV dissolved in the sterile essential medium, while the other half were orally administrated with 4 mL of the sterile essential medium. Then, the diarrhea of all piglets was observed for each day. Fecal consistency was scored twice daily at 0800 and 2000, as follows: 0, normal; 1, pasty; 2, semiliquid; and 3, liquid. The diarrhea mean cumulative score was calculated as [(Σ fecal scores for duration of PRV infusion)/n] [30].

Porcine RV preparation and virus titre determination (TCID_50_ value) were determined as described previously [11].

On the morning of d 19, following feeding at 1.5 h, all piglets were killed via intracardially injecting Na pentobarbital (50 mg/kg body weight) and jugular exsanguinations. Then, the intestine was removed, and the jejunum (proximal half of the small intestine) was quickly isolated, and flushed with ice-cold saline. The 3-cm segment of jejunum was fixed in 10% neutral-buffered formalin for analysis of histology. The jejunal mucosa was collected by scraping intestinal wall with a glass microscope slide, immediately frozen in liquid nitrogen, and stored at -80°C until analysis. Following pH measurement of cecal digesta with a pH meter (PHS-3C pH, Shanghai, China), approximately 3 g of cecal digesta were kept in sterile tubes, and immediately frozen at -80°C for microbial DNA analysis.

### Analysis of jejunal histology

The jejunal histology was determined as described previously [31]. Briefly, following the fixing, the jejunal segment was embedded in paraffin. And then, the consecutive section (5 μm) was stained with hematoxylin-eosin. The villus height and crypt depth of jejunal mucosa were determined at 40 × magnification with an Olympus CK 40 microscope (Olympus Optical Company).

### Analysis of NSP4, GLP-2, mucin 1 and mucin 2 levels in jejunal mucosa

The levels of NSP4 (Catalog No. YX-182214P) and GLP-2 (Catalog No. JM-E10007990) in jejunal mucosa were determined using the commercially available enzyme-linked immunosorbent assay (ELISA) kits from Nuoyuan Co., Ltd. (Shanghai, China) according to the manufacturer's instructions. The levels of mucin 1 (Catalog No. CSB-E15064p) and mucin 2 (Catalog No. CSB-E15066p) in jejunal mucosa were determined using the commercially available enzyme-linked immunosorbent assay (ELISA) kits from Cusabio Biotech Co., Ltd. (Wuhan, China) according to the manufacturer's instructions. The concentrations of NSP4, GLP-2, mucin 1 and mucin 2 were quantified with a BioTek Synergy HT microplate reader (BioTek Instruments, Winooski, VT), and the absorbance was measured at 450 nm.

### Bacterial DNA extraction and microbial real-time quantitative PCR

According to the manufacturer's instruction, bacterial DNA in cecal digesta was extracted with the Stool DNA Kit (Omega Bio-tek, Doraville, GA). The microbial real-time quantitative PCR was determined as described previously [11]. Briefly, the number of total bacteria was analyzed by real-time quantitative PCR using SYBR Premix Ex Taq reagents (TaKaRa Biotechnology (Dalian) Co., Ltd., Dalian, China) and CFX-96 Real-Time PCR Detection System (Bio-Rad Laboratories, Richmond, CA), and the number of *Lactobacillus*, *E. coli* and *Bifidobacterium* was analyzed by real-time quantitative PCR using PrimerScript^TM^ PCR kit (Perfect Real Time; TaKaRa Biotechnology (Dalian) Co., Ltd., Dalian, China) and CFX-96 Real-Time PCR Detection System (Bio-Rad Laboratories, Richmond, CA). All primers and probes, listed in Supplementary Table 2, were purchased by TaKaRa Biotechnology (Dalian) Co., Ltd. (Dalian, China). For the quantification of bacteria in test samples, specific standard curves were generated by constructing standard plasmids. Bacterial copies were transformed (log_10_) before statistical analysis.

### VFA analysis

The concentrations of VFA in cecal digesta were determined with a gas chromatographic method described by Chen et al. [17]. Briefly, the 1 g of digesta sample was thawed and suspended in 2 mL of distilled water in a screw-capped tube. Following vortexed, the sample was centrifuged for 10 min at 12000 g and 4°C. The supernatant (1 mL) was transferred into a tube containing 0.2 mL metaphosphoric acid. After standing for 30 min at 4°C, the tube was centrifuged for 10 min at 12000 g and 4°C. Aliquots of the supernatant (1 μL) were analyzed with a Varian CP-3800 gas chromatograph (Agilent Technologies, Santa Clara, CA).

### Analysis of antioxidant capacity

T-AOC and MDA concentration in jejunal mucosa were determined with commercial kits obtained from Nanjing Jiancheng Bioengineering Institute (Nanjing, China) and a UV-VIS Spectrophotometer (UV1100, MAPADA, Shanghai, China) according to the manufacturer's instructions.

### Western blot analysis

The antibody against ZO-1, occludin, claudin 1, claudin 3, p-mTOR (Ser^2448^), mTOR, Beclin 1, CHOP, Bax, Bcl-2 and β-actin were purchased from Cell Signaling (Davers, MA), Abcam (Cambridge, MA) and Santa Cruz Biotechnology Inc. (Santa Cruz, CA), respectively. Protein levels for the ZO-1, occludin, claudin 1, claudin 3, p-mTOR, mTOR, Beclin 1, CHOP, Bax, Bcl-2 and β-actin in jejunal mucosa were determined by Western Blot analysis as described previously [32, 33].

### Statistical analysis

All data, expressed as mean with their standard error, were analyzed as a 2 × 2 factorial with the general linear model procedures of the SAS (Version 8.1; SAS Institute, Cary, NC). The factors of models included the main effects of POS treatment (supplemented or not with POS in diets), RV challenge (infected or not with RV) and their interaction. When statistical significance was considered at *P* value less than 0.05, tendency at *P* value less than 0.10.

## SUPPLEMENTARY MATERIALS TABLES



## Abbreviations

RV: rotavirus
APOS: apple pectic oligosaccharide
ERS: endoplasmic reticulum stress
POS: pectic oligosaccharide
TCID_50_: Tissue culture infective dose 50
NSP4: non-structural protein 4
GLP-2: glucagon like peptide-2
ZO-1: zonula occludens 1
VFA: volatile fatty acid
T-AOC: total antioxidant capacity
MDA: malondialdehyde
p-mTOR: phosphorylated mammalian target of rapamycin
CHOP: CCAAT/enhancer-binding protein homologous protein
Bax: B-cell lymphoma/leukaemia-2-associated X protein
Bcl-2: B-cell lymphoma/leukaemia-2.
