# Supplementary material for: Dietary apple pectic oligosaccharide improves gut barrier function of rotavirus-challenged weaned pigs by increasing antioxidant capacity of enterocytes
Source: Oncotarget. 2017 Sep 28;8(54):92420–30. doi: 10.18632/oncotarget.21367 (PMC5696193; doi:10.18632/oncotarget.21367)
Supplement: Supplementary file 1 [file oncotarget-08-92420-s001.pdf]

Dietary apple pectic oligosaccharide improves gut barrier function of rotavirus-challenged weaned pigs by increasing antioxidant capacity of enterocytes

SUPPLEMENTARY MATERIALS

Supplementary Table 1: The composition and nutrient content of experimental diets

Items	Content	
	**CON**	**POS**
Ingredient composition, %		
Corn	29.33	29.33
Extruded corn	29.34	29.34
Fish meal	5.00	5.00
Whey powder	3.00	3.00
Corn starch	0.50	0.48
Apple pectic oligosaccharide	0.00	0.02
Soybean meal	11.00	11.00
Soybean protein concentrate	5.50	5.50
Extruded soybean	12.00	12.00
Soybean oil	2.00	2.00
L-Lysine-HCl	0.22	0.22
L-Threonine	0.06	0.06
DL-Methionine	0.12	0.12
Choline chloride	0.15	0.15
NaCl	0.30	0.30
$CaCO_3$	0.40	0.40
$CaHPO_4$	0.75	0.75
Vitamin premix[1]	0.03	0.03
Mineral premix[2]	0.30	0.30
Nutrient levels[3], %		
Digestible energy, MJ/kg	14.85	14.85
Crude protein	20.90	20.90
Total lysine	1.33	1.33
Total methionine and cysteine	0.71	0.71
Total tryptophan	0.21	0.21
Total threonine	0.74	0.74
Calcium	0.80	0.80
Phosphorus available	0.44	0.44

[1] Provided the following per kg of diet: Vitamin A, 9000 IU; Vitamin D_3, 3000 IU; Vitamin E, 20 IU; Vitamin K_3, 3.0 mg; Vitamin B_1, 1.5 mg; Vitamin B_2, 4.0 mg; Vitamin B_6, 3.0 mg; Vitamin B_{12}, 0.2 mg; Niacin, 30 mg; Pantothenic, 15 mg; Folic acid, 0.75 mg; Biotin, 0.1 mg.

[2] Provided the following per kg of diet: Fe (as $FeSO_4 \cdot 7H_2O$), 100 mg; Cu (as $CuSO_4 \cdot 5H_2O$), 6 mg; Zn (as $ZnSO_4 \cdot 7H_2O$), 100 mg; Mn (as $MnSO_4 \cdot H_2O$), 4 mg; Se (as $Na_2SeO_3 \cdot 5H_2O$), 0.3 mg; I (as KI), 0.14 mg.

[3] Calculated nutrient levels.

Supplementary Table 2: Primer and probe sequences used for real-time quantitative PCR

Bacteria	Nucleotide sequences (5'-3')	Annealing temperature (°C)	Product size (bp)
Bifidobacterium	Forward: CGCGTCCGGTGTGAAAG<br>Reverse: CTTCCCGATATCTACACATTCCA<br>Probe: ATTCCACCGTTACACCGGGAA	55.0	121
Lactobacillus	Forward: GAGGCAGCAGTAGGGAATCTTC<br>Reverse: CAACAGTTACTCTGACACCCGTTCTTC<br>Probe: AAGAAGGGTTTCGGCTCGTAAAA CTCTGTT	53.0	126
Escherichia coli	Forward: CATGCCGCGTGTATGAAGAA<br>Reverse: CGGGTAACGTCAATGAGCAAA<br>Probe: AGGTATTAACTTTACTCCCTTCCTC	55.0	96
Total bacteria	Forward: ACTCCTACGGGAGGCAGCAG<br>Reverse: ATTACCGCGGCTGCTGG	61.5	200
